# Key Dimensions for the Prevention and Control of Communicable Diseases in Institutional Settings: A Scoping Review to Guide the Development of a Tool to Strengthen Preparedness at Migrant Holding Centres in the EU/EEA

**DOI:** 10.3390/ijerph15061120

**Published:** 2018-05-30

**Authors:** Flavia Riccardo, Jonathan E. Suk, Laura Espinosa, Antonino Bella, Cristina Giambi, Martina Del Manso, Christian Napoli, Maria Grazia Dente, Gloria Nacca, Silvia Declich

**Affiliations:** 1 European Programme for Intervention Epidemiology Training (EPIET), European Centre for Disease Prevention and Control, (ECDC), Solna 169 73, Sweden; 2 Istituto Superiore di Sanità, Rome 00161, Italy; antonio.bella@iss.it (A.B.); cristina.giambi@iss.it (C.G.); martina.delmanso@iss.it (M.D.M.); christian.napoli@uniroma1.it (C.N.); mariagrazia.dente@iss.it (M.G.D.); gloria.nacca@iss.it (G.N.); silvia.declich@iss.it (S.D.); 3 European Centre for Disease Prevention and Control, (ECDC), Solna 169 73, Sweden; Jonathan.Suk@ecdc.europa.eu (J.E.S.); lau.espinosa.m@gmail.com (L.E.); 4 UCD School of Agriculture and Food Science, University College Dublin, Dublin D04 V1W8, Ireland; 5 Department of Medical Surgical Sciences and Traslational Medicine, “Sapienza” University of Rome, Rome 00185, Italy

**Keywords:** migrant health, preparedness, communicable diseases

## Abstract

Migrant centres, as other institutions hosting closed or semi-open communities, may face specific challenges in preventing and controlling communicable disease transmission, particularly during times of large sudden influx. However, there is dearth of evidence on how to prioritise investments in aspects such as human resources, medicines and vaccines, sanitation and disinfection, and physical infrastructures to prevent/control communicable disease outbreaks. We analysed frequent drivers of communicable disease transmission/issues for outbreak management in institutions hosting closed or semi-open communities, including migrant centres, and reviewed existing assessment tools to guide the development of a European Centre for Disease Prevention and Control (ECDC) checklist tool to strengthen preparedness against communicable disease outbreaks in migrant centres. Among articles/reports focusing specifically on migrant centres, outbreaks through multiple types of disease transmission were described as possible/occurred. Human resources and physical infrastructure were the dimensions most frequently identified as crucial for preventing and mitigating outbreaks. This review also recognised a lack of common agreed standards to guide and assess preparedness activities in migrant centres, thereby underscoring the need for a capacity-oriented ECDC preparedness checklist tool.

## 1. Introduction

Migration, one of the determinants of population change in the European Union (EU), is a continuous long-term reality [1] that has been described also as a driver for economic growth [2]. Notwithstanding, it is often addressed in a complex climate, where security, rather than health, drives management priorities [3]. In this context, EU public health authorities are asked to provide relevant, proportionate, and targeted action [4]. In 2015, Europe received over 1.2 million asylum seekers [5], placing considerable strain on the provision of health services [6].

Undocumented migrants entering continental Europe are often placed for a short period of time (hours–days) in temporary accommodation facilities. Here, they are identified, offered an initial medical assessment/screening [7], and provided medical care, if needed. Following this, depending on their legal status (asylum seekers vs irregular migrants), they can be transferred to medium term accommodation facilities, migrant reception or detention institutions (hereby migrant centres), where they can stay for a longer period of time (weeks–months). 

Migrant centres in Europe are institutions that typically host semi-open (reception centres) and closed (detention centres) communities. As stated by Basu et al., institutions “*are characterized by a combination of multiple and interacting social determinants of infectious disease spread (…) including close and prolonged human contact, poor ventilation, containment of highly susceptible or immunocompromised groups, and significant flows of persons into and out of these institutions*” [8]. It can be expected that, as in other institutional settings, like prisons, military barracks, and schools, migrant centres face specific challenges in preventing and controlling disease transmission. Dimensions such as human resources, medicines/vaccines, sanitation/disinfection, and physical infrastructure are all critical for the prevention, early detection, and outbreak management in institutional settings. It is, however, uncertain what could be priorities for action in migrant reception/detention centres. 

In 2016, the European Centre for Disease Prevention and Control (ECDC) developed a “Preparedness checklist tool to strengthen preparedness against communicable disease outbreaks at migrant reception/detention centres” [9]. This tool guides the assessment of gaps in critical dimensions within these centres, and could be useful for decision makers in EU/EEA member states to prioritise investments to enhance routine capacity, as well as preparedness for large sudden influxes of migrants. 

The development of this tool was evidence-based, according to a scoping review of the scientific and grey literature. There were two key objectives to this review. The first was to identify challenges for the prevention and control of communicable diseases in migrant centres and similar institutional settings (hereafter objective 1). The second was to identify suitable approaches for assessing strengths and weaknesses in controlling/preventing communicable diseases in migrant centres (hereafter objective 2). This paper presents the results of this scoping study and how those findings impacted the ECDC tool development.

## 2. Materials and Methods 

Studies in peer-reviewed journals and grey literature were included if:

(i) they were published between January 2000 and October 2015 in English, French, or Italian;
(ii) they referred to institutional settings (i.e., institutions hosting “closed” or “semi-open” communities);
(iii) they described types of disease transmission in these settings, and which dimensions were more frequently critical for transmission prevention/outbreak control (so as to address objective 1);
(iv) they described existing assessment tools or prior assessments (so as to address objective 2).

Articles/reports were excluded if they did not address human health and when abstracts/full texts were not retrievable from open source and journal subscriptions available to the Italian Institute of Public Health (ISS) and ECDC. The selection process followed four phases: identification, screening, eligibility, and inclusion, as described in the PRISMA statement [10].

We defined four axes: Exposure, Population, Outcome, Methods, to develop a set of common search roots for the scientific literature search. We identified Medical Subject Headings (MeSH) terms for each search axis, unless term definitions were unrelated to the search context. 

The exposure axis included search terms to identify articles on disease transmission; population included terms on institutional settings and methods included terms on assessment tools or prior assessments. The outcome axis included search terms on prevention and control, and on emergency preparedness. 

**Figure 1:**
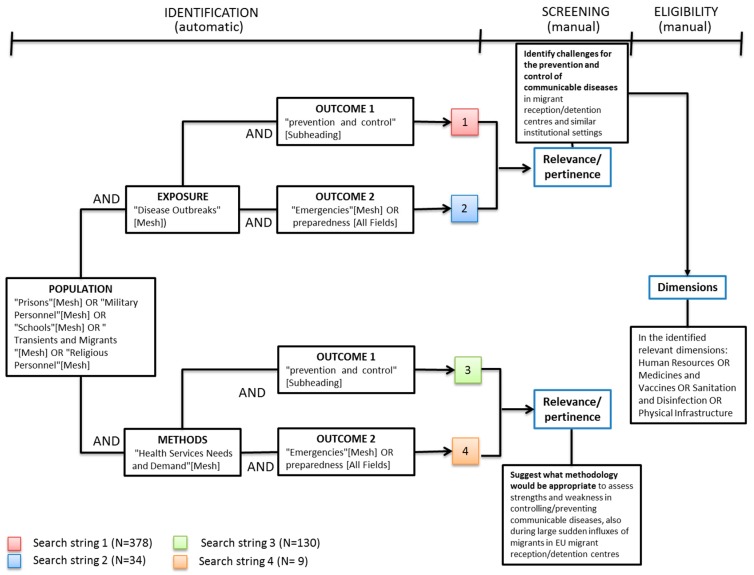
Scientific literature search strategy diagram. EU: European Union.

Four search strings were developed by combining the search terms in each axis (Figure 1), systematically giving preference to terms and combinations that provided a greater article yield. Articles were extracted from PubMed on 18 October 2015. 

The same four search axes guided the grey literature search. Articles were “hand-searched” for potentially relevant grey literature from October to December 2015 on the following targeted websites: ECDC, World Health Organization (WHO), the International Organization for Migration (IOM), United Nations High Commissioner for Refugees (UNHCR), Doctors Without Borders (MSF), the Red Cross, the UN Office for the Coordination of Humanitarian Affairs (OCHA).

One reviewer screened each article/report for relevance, to identify which ones would then be analysed in full text, on the basis of the abstracts (scientific literature) and the executive summaries (grey literature). Thereafter, in the eligibility phase, selected articles/reports were analysed in full text to identify if they addressed objective 1 or 2 (or both). For articles addressing objective 1, we also documented which dimensions for communicable disease prevention/control were identified to be critical (dimension analysis). 

A dimension was classified as critical when its inadequacy was recognised by authors as one of the causes of outbreak development, or when its reinforcement through response actions was recognised as pivotal for successful outbreak containment. There were four pre-identified dimensions (human resources, medicines/vaccines, sanitation/disinfection, and physical infrastructure). Additional dimensions were documented when identified as critical in the literature (hereby additional dimensions).

## 3. Results

### 3.1. Selection Process

**Figure 2:**
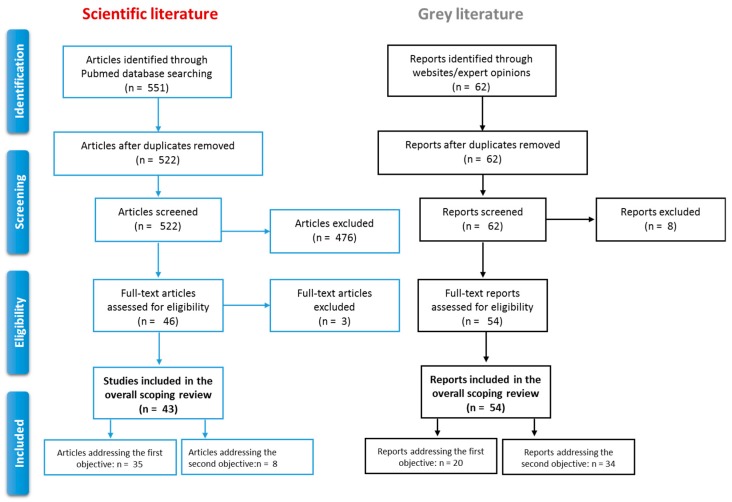
Results of the scientific and grey literature scoping review.

We identified 551 scientific articles, of which 522 were screened, and 46 assessed in full text. Three articles were excluded in the eligibility phase because they did not report on actual disease transmission but on mathematical models, and on options to design health policies and strategies (Figure 2). Among the 43 included scientific articles, 72% focused on European/North American countries, and 49% were published after 2010. Most articles (86%) focused on educational institutions (schools, universities, college etc., including both day and boarding institutions) and correctional settings (jails and prisons). Two papers targeted migrant centres [11,12]. Most articles (35) addressed the first objective, the remaining addressed the second. 

We retrieved 62 grey literature reports and included 54 in the review (Figure 2). Twenty addressed the first objective. All were situation analysis studies focusing on European migrant centres and most (80%) were produced by the WHO PHAME [13] and the IOM EquiHealth Projects [14] between 2013 and 2015. The remaining 34 reports addressed the second objective.

### 3.2. Challenges for the Prevention and Control of Communicable Diseases in Migrant Centres and Similar Institutional Settings

Fifty-five articles/reports addressed the first objective of this review (35 peer reviewed articles and 20 reports—see Supplementary File 1 for further details). Of those, 27% were published in the Unites States, 20% by WHO, and 11% by IOM. The 35 articles focused on what favoured outbreak development or its control in a wide range of institutional settings from an operational standpoint. Conversely, the 20 reports addressing the same objective focused exclusively on national migrant reception systems and facilities in Europe, generally from a wider public health perspective [15,16,17,18,19,20,21,22,23,24,25,26,27,28,29,30,31,32,33,34]. 

**Figure 3:**
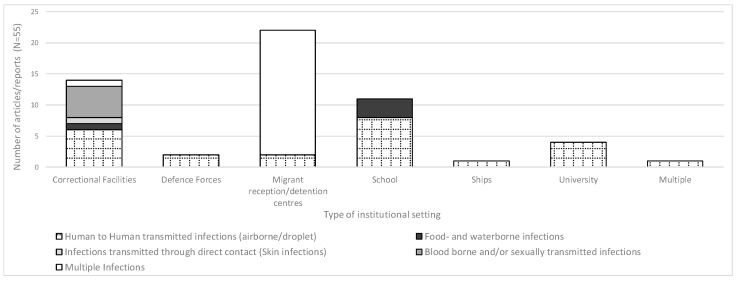
Number of articles or reports addressing the first review objective (*n* = 55), by type of infectious disease transmission and setting.

We found evidence of both direct and indirect transmission of gastrointestinal infections [35,36] in both educational and correctional institutions (Figure 3). In a literature review of reports of gastrointestinal outbreaks in correctional settings, authors found that “*Bacterial agents were associated with 76% of outbreaks, and viral agents were associated with 21%. One outbreak was associated with the protozoan parasite Cryptosporidium, while ‘multiple organisms’ were associated with an additional outbreak (…) Routes of transmission (…) were foodborne in 67% of cases and person-to-person in 11% of cases*” [36]. Outbreaks of human-to-human transmitted infections were described in different institutions where close physical congregation of individuals occurs [11,37,38,39,40,41,42,43,44,45]. Conversely, outbreaks of skin infections [46], sexually transmitted diseases (STD), and blood borne viruses (BBV) [47,48,49] were described more frequently in correctional settings: “*High syphilis prevalence and multiple sexual partnerships result in the potential for extensive syphilis transmission. Condoms are not likely used*” [47]. Some studies have identified factors pointing to an increased risk of transmission among inmates: “… *disproportionate incarceration of people at higher risk for HIV infection, (…) persons with mental illness, substance users, those who trade in sex (…) inmate behaviours that risk HIV transmission—including sex (forced and consensual), injection drug use and tattooing—and the limited availability of condoms and clean needles*” [49]; “*…many prison entrants have histories of injecting drug use (IDU), and thus already have high prevalences of blood borne viruses (BBVs). (…) … the lack or under-supply of preventive measures (…) combined with extreme social conditions, and consequent prisoner behaviour, creates extra opportunities for BBV transmission. Viruses such as HIV can be transmitted sexually, through sharing injecting drug equipment, by non-sterile tattooing, and transmission of blood or bodily substances during assaults (…) sharing of equipment for shaving and haircutting …*” [48].

The articles/reports focusing specifically on migrant centres described mainly generic/multiple possible disease exposures and, when specified, the types of transmissions more frequently described were human-to-human and/or via contaminated water/food [11,12,20]. 

Specific challenges in preventing and controlling infectious disease spread have been described in correctional settings. In addition to more general challenges in managing health issues within secure environments [50,51], we found evidence that dispersed correctional systems that combine rapid turnover (jails) and longer term (prisons) detention facilities, with frequent interfacility transfers, influence disease transmission dynamics. Rapid turnover creates an inflow of people in rapidly consecutive cohorts (a “revolving doors” effect [52]). An inflow of susceptible people within a closed or semi-open community experiencing an outbreak, has been shown to slow the creation of herd immunity and can act as a transmission amplifier [53,54], while interfacility transfers can facilitate disease spread: “*Contacts occurred during inmate transports between prisons, at a courthouse, and within the prisons* (…) *Prisons and prison transport vehicles are crowded environments that create potential for the spread of respiratory and other infections including measles, rubella, chickenpox, tuberculosis and meningococcal disease. The transport system that supports a devolved correctional system, sets this environment apart from other crowded environments such as boarding schools, and aligns it with aspects of military camps*” [55]. All this, combined with factors associated with living conditions, can favour infectious disease transmission: “*Detainees are more likely to become infected as a result of significant overcrowding in prisons, poor living conditions, poor nutrition, and physical and emotional stress*” [52]. Finally, it should be noted that “*If an individual is in a correctional institution, the primary purpose of the setting are custody and confinement. Although healthcare is mandated, it is not the priority of custody institutions*” [51]*.*

**Figure 4:**
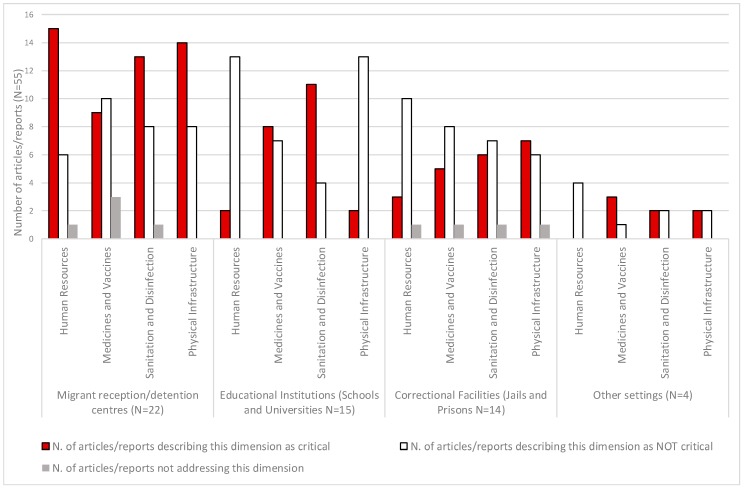
Number of articles or reports addressing the first review objective (*n* = 55), by dimension and institutional setting.

Overall, sanitation/disinfection was described as critical in 32 articles/reports (58%), followed by medicines/vaccines, physical infrastructure (25 studies/reports; 45% each), and human resources (20; 36%). According to the type of institutional setting, different dimensions were more frequently described as critical. Sanitation/disinfection and medicines/vaccines were more frequently described as critical in articles/reports focusing on educational institutions, while physical infrastructure was the only dimension more frequently described as critical in articles/reports on correctional facilities. Among the 22 articles/reports that specifically focused on migrant reception/detention centres, three dimensions were more frequently described as critical: human resources (15 articles/reports, 68%), physical infrastructure (14 articles/reports, 64%), and sanitation/disinfection (13 articles/reports, 59%). For example, site visits documented that: “*… suboptimal living conditions, staff numbers and skill mix in detention centres and in open centres are major concerns. Unhygienic surroundings, and in particular toilets, pose further health risks for migrants*” [18]; and, further: “*Unfortunately, the facilities identified as migrant centres were in very poor condition, lacking electricity, heating or proper sanitation systems. Essential services such as food and health care were not systematically delivered”* [28]; “*The infrastructure and the living conditions in reception, refugee, and detention centres vary from one facility to another. The most frequent problem is budget deficit, which affects living conditions (i.e., poor diet, overcrowding, excessive cold or heat, inadequate sanitation, lack of social activities)* [34]*”*. Shortages in availability of medicines/vaccines were described less frequently (9 studies/reports, 41 %) (Figure 4).

The most frequently identified additional dimension in both articles and reports was overcrowding (24 articles and reports, 55%). The majority (15, 68%) of all articles and reports on migrant facilities reported overcrowding to be an issue.

**Figure 5:**
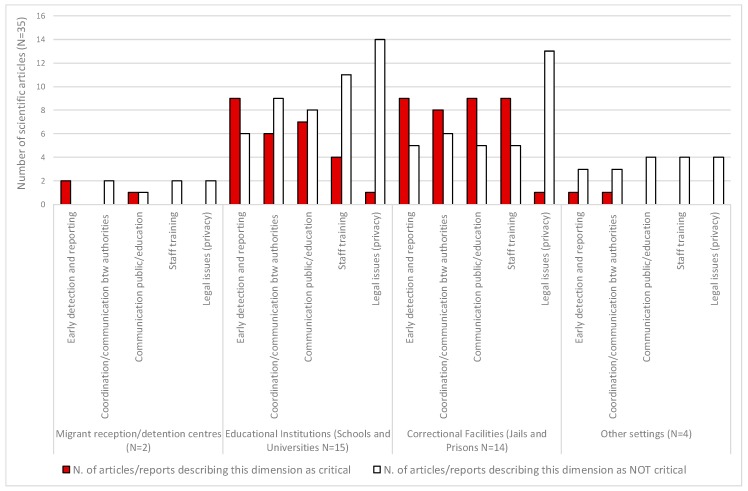
Number of scientific articles addressing the first review objective (*n* = 35), by additional dimension and institutional setting.

Among the 35 peer-reviewed articles, five other aspects were also described recurrently as critical. Foremost were early detection and reporting (21 studies, 60%) followed by communication with, and education of, the public (17 studies, 49%), coordination between authorities (14 studies, 40%), staff training (9 studies, 26%), and management of legal/ethical issues (2 studies, 6%). Among those, as shown in Figure 5, early detection and reporting was more frequently described as critical, rather than not critical, in articles/reports focusing on migrant, educational, and correctional settings. Coordination, communication, and staff training emerged as more frequently critical only in articles/reports on correctional facilities. 

**Figure 6:**
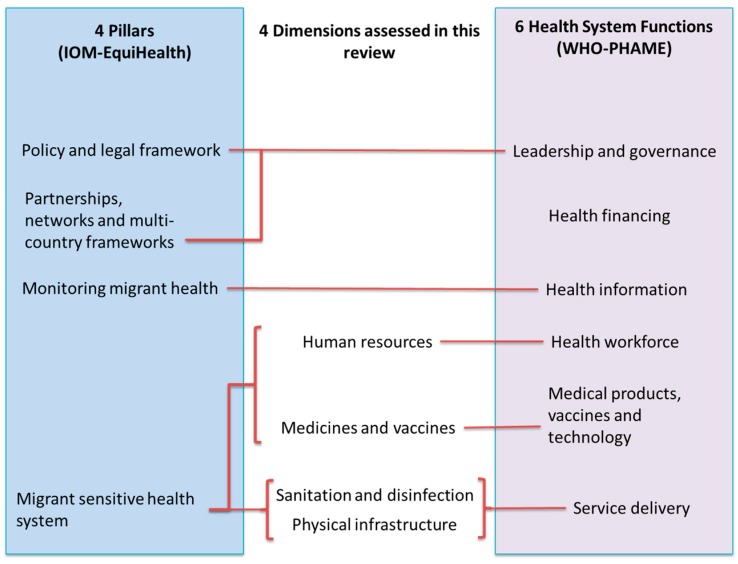
Assessment frameworks used in the International Organization for Migration (IOM) and World Health Organization (WHO) situation assessments in relation to the scoping review dimensions.

The pillars and functions considered by IOM and WHO in the 20 situation analysis studies included in this review only partly matched the dimensions that we had identified (Figure 6). Among those, *health information* (in particular, focusing on surveillance and communication systems) and *health financing* were found by WHO and IOM as recurrently critical within migrant centres. Both did not clearly overlap, neither with the dimensions we had defined before the review, nor with the additional dimensions we identified by analysing the included articles/reports.

### 3.3. Suitable Approaches for Assessing Strengths and Weaknesses in Controlling/Preventing Communicable Diseases

Forty-two articles/reports addressed the second objective of this review. These included 34 grey literature reports (9 tools and 25 risk/needs assessments and guidance documents) and 8 scientific articles.

The nine tools [56,57,58,59,60,61,62,63,64] we analysed were principally checklist-based (See Supplementary File 1 for further details). Only one was a self-assessment tool [58]. WHO tools were mainly aimed at assessing health systems [56,59], but also included instruments targeting hospital administrators and emergency managers [60]. Other tools included instruments to assess refugee/displaced population emergencies [61], conduct health needs assessments in prison settings [63,64], or support European parliamentarians visiting immigration detention centres [62]. In addition, while some were clearly oriented to emergency preparedness [56,59,60], another adopted a capacity assessment approach [57] in the framework of the International Health Regulations (IHR) [65]. 

The methods proposed in the eight scientific articles were more diverse, including mathematical models, the development of a risk assessment tool, a table top exercise, training, the review of preparedness plans, surveys, and a stakeholder analysis. 

The 25 risk/needs assessments and guidance documents used different reference standards to measure adequacy within migration holding centres with respect to the dimensions we explored. For example, documents from MSF [61,66], WHO [57,60,67], and UNHCR [68] were cited as standard references in different ECDC documents [15,31,69] included in this review. UNHCR [70] quoted a tool designed to identify strategic humanitarian priorities [71] and the Humanitarian Charter and Minimum Standards in Humanitarian Response (Sphere Project) [72]. Among three documents on migrant holding facilities in Italy: the first document, a WHO situation analysis [21], quoted UNHCR [73] and the Sphere Project; the second document, of the Italian Red Cross [74], quoted a European Red Cross guideline [75]; and the third document, issued by the Veneto Region [76], referred to a WHO guidance [67].

## 4. Discussion

Disease transmission has been repeatedly documented in institutional settings, including migrant facilities. Human-to-human transmitted and food and waterborne infections have been described in a variety of institutions, while skin infections have been described in correctional and migration settings. These findings are consistent with the results of syndromic surveillance in migration centres in Italy [77,78] and Greece [79,80], that have identified signs/symptoms of scabies, respiratory tract infections, and gastrointestinal infections as the most frequently reported syndromes.

Human resources, medicines/vaccines, sanitation/disinfection, and physical infrastructure were all identified as dimensions to consider for the prevention/control of communicable diseases in migration centres. Among those, we found human resources, physical infrastructure, and sanitation to be most frequently identified as critical. Although cultural mediators are considered essential to provide culturally competent services [81], they were recurrently described as insufficient or unavailable within migrant centres. Poor physical infrastructures, poor environmental hygiene conditions, and a lack of clean clothing, bedding, and personal hygiene equipment were also recurrent challenges [18,28], particularly during large sudden influxes of migrants. However, this was not always the case [34]. The quality of infrastructure/sanitation levels among different migrant holding centres can vary, also within the same country [19]. Challenges in the physical infrastructure of migration facilities might be more evident than in other institutional settings, because surges in migration can rapidly make infrastructures inadequate to host larger numbers of people than initially intended. Conversely, we found medicines/vaccines more often mentioned as critical in educational and other institutional settings. This finding is partly due to the fact that several studies focused on the 2009 pandemic influenza, and largely discussed the use and timing of vaccination and/or antiviral treatments in educational institutions. 

This review was conducted embracing a wide range of institutional settings, due to the lack of comprehensive studies with a specific focus on migrant centres and communicable disease transmission and control. The general assumption behind this choice was that a broad spectrum of possible mechanisms drives communicable disease transmission and challenge outbreak control in institutional settings hosting closed or semi-open communities, like migrant centres, and that lessons learned could be translated across settings with similar challenges. We found documents related mainly to three types of institutions: migrant centres, educational institutions, and correctional facilities. 

The documents we reviewed with a focus on educational institutional settings described different organisation systems and challenges compared with what could be expected in migrant reception (semi-open) centres. Coherently, the pattern of the most frequently described critical dimensions was different, with human resources and physical infrastructure (the most frequently critical dimensions in migrant centres) identified more frequently as not critical in educational settings. 

Conversely, this review highlighted that specific communicable disease transmission dynamics challenge dispersed correctional systems. This is an interesting finding because migration reception in many EU countries is organised in a similar way, with high turnover short-term facilities and reception/detention institutions designed to host migrants for longer periods. We concluded that evidence and experience on communicable disease prevention and control in dispersed correctional settings might be something to consider/assess also in relation to similarly dispersed migrant reception systems. These considerations guided the selection of which key dimensions identified in this review to include in the ECDC preparedness checklist tool to strengthen preparedness in migrant centres [9].

In particular, overcrowding, coordination, health information, and health financing were all dimensions that had not been defined prior to conducting this review. *Overcrowding* emerged as frequently critical, in particular, in migrant centres, and was therefore naturally included in the tool as an element that could increase the risk of outbreaks. The need to develop functional *coordination* among the different actors was instead highlighted, in particular, in dispersed correctional settings. Nonetheless, we included also this aspect in the tool because, like those institutions, migrant detention/reception centres are hubs of many different actors working within and outside the centre itself. *Early detection and reporting* was mentioned as critical in articles on almost all the settings explored and *health information* was a recurring critical element in the WHO/IOM situation analysis reports. This finding is in line with the outcome of an ECDC expert opinion [15] that recommended the implementation of syndromic surveillance systems in migrant centres. ECDC has subsequently developed a “Handbook on implementing syndromic surveillance in migrant centres and other refugee settings” [82], so this aspect was included also in the checklist tool. 

Finally, we found that the literature stresses the importance of *health financing* as a relevant dimension in institutional settings. Lack of sustained funding can explain the lack of human resources, stock ruptures in all types of commodities, including pharmaceuticals, inadequate infrastructure, maintenance, and hygiene/sanitation levels. In relation to migration, several EU governments are highly dependent on EU project and emergency funds in facing sudden influxes of migrants [25,31], while in some member state NGOs and international organisations have been supporting national governments by providing services within migrant centres. Thus, health financing sustainability has been proposed as an indicator of the fragility of migration emergency response systems in terms of their viability and surge capacity. For this reason, this aspect was also included in the ECDC checklist tool. 

Through this review, it was concluded that common standards and reference tools for the assessment of needs and requirements in EU migrant centres are currently lacking and that the number of studies particularly addressing this topic is still limited. Consequently, the possibility of designing a tool against a pre-established set of standards was precluded. 

In conclusion, based upon this review, ECDC developed a checklist tool intended for EU/EEA public health authorities who need to self-assess the capacity for communicable disease prevention and control at reception/detention centres hosting migrants for weeks/months (medium-term) in order to identify gaps and set priorities for development. Its aim is to monitor and support capacity development to prevent the onset, and improve the management of, communicable disease outbreaks at medium-term migrant reception/detention centres, both on a day-to-day basis and in the event of a sudden influx of migrants. The tool aims to assess capacity based on three general objectives:

- Outbreak prevention (covering communicable disease prevention, rapid case detection, and case management)
- Outbreak control (covering outbreak detection and control in the reception/detention centre being assessed)
- Outbreak management during a large sudden influx of migrants (communicable disease prevention, detection, and control during a large sudden influx of refugee migrants at the reception/detention centre being assessed.

One of the first elements adopted from this scoping review was the identification of the appropriate scope for the tool. The tool assesses preparedness capacity in relation to the medium-term accommodation of migrants within centres, thereby complementing an existing tool developed by the WHO PHAME project analysed in this study. 

The second general element adopted from the scoping review was to choose a methodological approach that would not assess against a common recognised set of standards, that we found to be missing, but that would be based on capacity, using a health system strengthening approach. Therefore, the tool refers to the International Health Regulations (IHR) as a framework, focusing on capacity development. In terms of methodology, the WHO Assessment Tool for Core Capacity Requirements at Designated Airports, Ports, and Ground Crossings was taken as a model and adapted to the context of medium-term migrant reception/detention facilities.

Finally, the scoping review defined the dimensions to include in the tool, both confirming the relevance of pre-defined ones and incorporating novel ones. In its final version, the tool addressed the following: human resources; medicines and vaccines; physical infrastructure; sanitation; health financing; coordination; health information; overcrowding. A total of 94 statements for self-assessment were designed to cover the objectives of the tool and address all the identified dimensions [9].

Given the broad spectrum of possible mechanisms driving challenges in communicable disease transmission in institutional settings hosting closed or semi-open communities, and the dearth of literature specifically focusing on outbreak prevention and control in migrant centres, we chose to adopt a very broad approach for this review. This approach has intrinsic limitations, due to the diversity of “institutional settings” considered and, within the migration hosting system, the diversity of reception and detention centres in terms of conditions and public health implications. For this reason, the scoping review approach was chosen, as this kind of review allows for the identification of possible issues, even if it will not give a systematic quantification of effects. A scoping review, as described by Arksey and O’Malley [83], while systematic in its collection of data, as opposed to traditional systematic reviews, tends to address broader topics where many different study designs might be applicable, and is less likely to seek to address very specific research questions nor, consequently, to assess the quality of included studies. This type of rapid review might not describe research findings in any detail, but is a useful way of mapping fields of study where it is difficult to visualise the range of material that might be available. The choice of not quantifying effects is also justified because the contribution of each driver to obstacles in communicable disease prevention/control is setting-specific, while the underlying mechanisms can be common across settings with common characteristics. Further, results were stratified by the type of institution, and discussed separately to highlight when data was retrieved directly on migration hosting facilities and when it was evidence originated from other settings that might be relevant also to the migration hosting system. As a result of this methodological choice, limiting the search to PubMed was considered adequate.

While this scoping review study used wide search terms and a long-time frame, we chose to limit our scientific search to articles in English, Italian, and French language. The language choice was guided by the language abilities of the reviewer and led to the inclusion of two globally spoken languages (English, French) and of Italian, the language spoken by one of the EU countries mostly affected by the recent migration crises in the region. 

Further, not including other MeSH terms (such as “refugees”) limited to our ability to identify articles. The impact of this specific aspect was assessed, and found to be contained (including the term refugee would have led to a non-deduplicated increase of 12% in the number of abstracts. The reason lies in the fact that articles were also captured by the use of the term “migrants and transients”, that was included. 

A single reviewer was engaged in reading and analysing abstracts and full-text articles/reports, and this could have led to a subjective collection of data. However, the information collected, e.g. if an aspect was mentioned as critical or not, was selected to be as simple and less prone to subjective assessment as possible, and standardised as much as possible in the study protocol to limit any negative impact this choice could have had.

We were limited in our grey literature search to our knowledge of relevant institutions and websites. We considered this not to hinder the general aim of the review that was not to comprehensively assess literature in relation to an intervention, but rather, to gather a general understanding of disease transmission drivers in institutional settings, identify more frequent critical dimensions for outbreak prevention/control, and types of tools that could be adapted to a migrant setting. 

## 5. Conclusions

As discussed, this literature review has looked across different institutional settings to identify the foundations for the development of the ECDC preparedness checklist tool for strengthening preparedness at migrant reception/detention centres [9]. This study enabled us to confirm the need and shape the structure of the tool, identifying human resources, medicines/vaccines, sanitation/disinfection, physical infrastructure, overcrowding, coordination, health information, and health financing as important dimensions for prevention/control of communicable diseases in migration centres. Furthermore, this study highlighted how evidence and experience on communicable disease prevention and control in dispersed correctional settings might be something to consider/assess, also in relation to similarly dispersed migrant reception systems. Moving forward, it will be important to pilot test this tool in field settings, and to generate a broad dialogue aimed at identifying common standards that migrant holding centres could aim to achieve in European settings.

## Supplementary Materials

The following are available online at http://www.mdpi.com/1660-4601/15/6/1120/s1, Supplementary File 1: Database supplementary file.
